# Hypoalbuminemia During the First 24 Hours Is Not an Independent Risk Factor for Mortality in Major Burns

**DOI:** 10.1093/jbcr/irag050

**Published:** 2026-04-09

**Authors:** Christoph Ils, Tobias Zingg, Mette M Berger, Jean-Daniel Rouvé, Lara Montoro, Anthony de Buys Roessingh, Karim Al-Dourobi, Yves Harder, Olivier Pantet

**Affiliations:** Department of Intensive Care Medicine, Lausanne University Hospital and University of Lausanne, 1011 Lausanne, Switzerland; Department of Visceral Surgery, Lausanne University Hospital and University of Lausanne, 1011 Lausanne, Switzerland; Faculty of Biology and Medicine, University of Lausanne, 1011 Lausanne, Switzerland; Department of Anesthesiology, Lausanne University Hospital and University of Lausanne, 1011 Lausanne, Switzerland; Faculty of Biology and Medicine, University of Lausanne, 1011 Lausanne, Switzerland; Department of Pediatric, Children and Adolescent Surgery Service, Lausanne University Hospital and University of Lausanne, 1011 Lausanne, Switzerland; Department of Plastic Reconstructive and Aesthetic Surgery and Hand Surgery, Lausanne University Hospital and University of Lausanne, 1011 Lausanne, Switzerland; Department of Plastic Reconstructive and Aesthetic Surgery and Hand Surgery, Lausanne University Hospital and University of Lausanne, 1011 Lausanne, Switzerland; Department of Intensive Care Medicine, Lausanne University Hospital and University of Lausanne, 1011 Lausanne, Switzerland

**Keywords:** burn injury, albumin, hypoalbuminemia, mortality, critical care

## Abstract

Major burn injuries commonly present with hypoalbuminemia within the first 24 h. This study aimed to determine whether early hypoalbuminemia independently predicts mortality in severe burns. This single-center, retrospective cohort study included patients aged 14 years and older with burns covering at least 20% of their body surface area, admitted between January 2006 and December 2023 to a university teaching hospital. Exclusion criteria were admission more than 8 h postinjury or transfer to another unit within the first week. Albumin levels within the first 24 h were recorded. The primary outcome was 28-day mortality. A total of 161 patients were included with a median burn area of 38% (IQR 25-65) and an Abbreviated Burn Severity Index score of 9 (IQR 7-11.5). Mortality was 33% (53/161). While univariate analysis showed that lower albumin was associated with increased mortality (OR 0.91; 95% CI, 0.86-0.96; *P* = .001), this association was not significant after adjustment for burn severity (OR 0.99; 95% CI, 0.93-1.06, *P* = .87). The predictive value of minimum serum albumin for mortality was low with an area under the curve of 0.68. The optimal albumin threshold for predicting mortality was 24 g/L (sensitivity 81.0%, specificity 49.5%). Albumin levels below this threshold were not significantly associated with higher mortality in a time-to-event analysis (HR 1.72; 95% CI, 0.79-3.73, *P* = .169). Hypoalbuminemia in the first 24 h was not found to be an independent risk factor for 28-day mortality in this cohort, suggesting it as a marker of burn severity rather than an independent predictor.

## INTRODUCTION

Patients with severe burn injuries requiring the intensive care unit (ICU) are a complex subtype of critically ill patients. When thermal burn injuries exceed 20% of TBSA, the patients develop shock. The pathophysiology involves a rapid increase in capillary permeability and glycocalyx shedding^1^ driven by massive liberation of inflammatory mediators (eg, histamine and chemokines). This cascade leads to fluid extravasation into the interstitial space, proportional to burn size, causing distributive and hypovolemic shock.^2^ Concurrently, the stress response and catecholamine release increase systemic vascular resistance.^3^ Plasma extravasation and leakage of proteins, including albumin, from the intravascular to the interstitial space exacerbates edema formation.^2^ Fluid resuscitation is essential to prevent organ dysfunction and death in burn shock. While isotonic crystalloids form the cornerstone of resuscitation, strict adherence to the Parkland formula has been associated with important “fluid creep” and over-resuscitation complications.^4^ Consequently, hyperpermeability and crystalloid administration frequently result in low serum albumin levels in patients with severe burn injuries.^5–11^

Hypoalbuminemia is consistently associated with mortality in nonburn critically ill patients, a finding recently reaffirmed.^12^ The pathophysiology of hypoalbuminemia in major burns may differ. Several studies have reported an association between hypoalbuminemia during the first 24 h and increased mortality in patients with burn injuries, with some authors proposing it as an independent risk factor.^5–11^ This has supported the controversial use of colloids, including albumin, for burn resuscitation.^13^^,^^14^ Physiologically, very low albumin levels lead to a steep decrease in plasma oncotic pressure, potentially worsening fluid shifts.^15^ Due to the persistent controversy, the American Burn Association (ABA) guidelines have historically recommended delaying albumin administration until 12 h postinjury.^13^

Given that the link between early hypoalbuminemia and mortality remains unestablished, we hypothesized that low serum albumin during the first 24 h might be an independent predictor of mortality in patients with severe burn injuries.

## MATERIALS AND METHODS

### Study design and eligibility

A single-center retrospective cohort study was conducted in the burn ICU at a university teaching hospital in Switzerland. The records of patients with burn injuries admitted between January 2006 and December 2023 were reviewed for eligibility. Inclusion criteria were age ≥ 14 years and a TBSA of at least 20%. Exclusion criteria were admission > 8 h after the injury and transfer to another burn unit or hospital during the first week. Patients who died within the first 24 h were not excluded, as our policy is to provide maximal care for at least 48-72 h before considering withdrawal of treatment.

The study was approved by the ethics committee (CER-VD, BASEC ID 2018-02268). Consent forms were sent and checked for responses on November 18, 2024. Patients were considered eligible if the consent form was signed and accepted, if the patient was deceased with unknown consent status or if the patient was alive with written consent forms sent 3 times at different time intervals without any response (Article 34 of the Federal Act on Research involving Human Beings, RS 810.30).

Patients were treated according to the institutional resuscitation protocol: TBSA was assessed by the rule-of-nines. The initial target fluid volume for resuscitation was 2-4 mL/kg/% and then titrated according to diuresis or advanced hemodynamic monitoring. Albumin administration was authorized after the eighth hour postadmission. Inhalation injuries were systematically sought via direct examination and bronchial fibroscopy. From 2012 to 2023, the criteria of Ikonomidis et al.^16^ were applied and replaced, since 2023, by the international RAND/UCLA expert panel criteria.^17^

### Data collection

Data were extracted from electronic medical records using internal software systems: MetaVision (iMDsoft), Soarian (Siemens), and Archimede (archiving platform).

Collected variables included age, sex, TBSA, full-thickness burns, inhalation injury, Simplified Acute Physiology Score II (SAPS II), Abbreviated Burn Severity Index (ABSI) scores, preadmission weight, duration of mechanical ventilation and ICU length of stay. Other data included daily serum albumin levels, time to first dose of albumin, volume of crystalloid, albumin, or other transfusion products administered, amount of noradrenaline administered, urinary output, serum creatinine levels, renal replacement therapy (RRT), and 28-day mortality. The minimum values for albumin levels in the first 24 h were reported. The time frames of measurement were recorded: 0-8, 8-16, and 16-24 h.

Mortality was measured at 28 day. The vital status and date of death of patients were verified using the Swiss death registry. Acute kidney injury was defined according to the Kidney Disease Improving Global Outcomes (KDIGO) classification,^18^ as a KDIGO stage ≥ 1, based on the worst creatinine values during the first 7 days of hospitalization and assuming the first creatinine values to be baseline. The need for RRT during the hospitalization was also reported.

### Statistical analyses

Statistical analyses were performed using Stata (v18.0; *StataCorp LLC,* College Station, TX, USA). Data distribution was tested using the Shapiro–Wilk test. The Wilcoxon signed-rank test and Student’s *t*-test were used to compare quantitative variables. Pearson’s chi-square test and Fisher’s exact tests were used to compare qualitative variables. A logistic regression model was used to determine if serum albumin levels were correlated with 28-day mortality. To determine independence from confounding factors, the model was adjusted for the ABSI score, which integrates age, sex, TBSA, and presence of full-thickness burn. Receiver operating characteristic curve analysis tested the best threshold values (using the Youden index) of albuminemia and the area under the curve (AUC) was calculated. Time to-event analyses were estimated with Kaplan–Meier and Cox analysis. A 2-sided *P* < .05 was considered statistically significant. Quantitative/continuous variables are expressed in median and interquartile range (IQR) whereas qualitative/categorical variables are expressed as counts (*n*) and percentages.

## RESULTS

**Figure 1:**
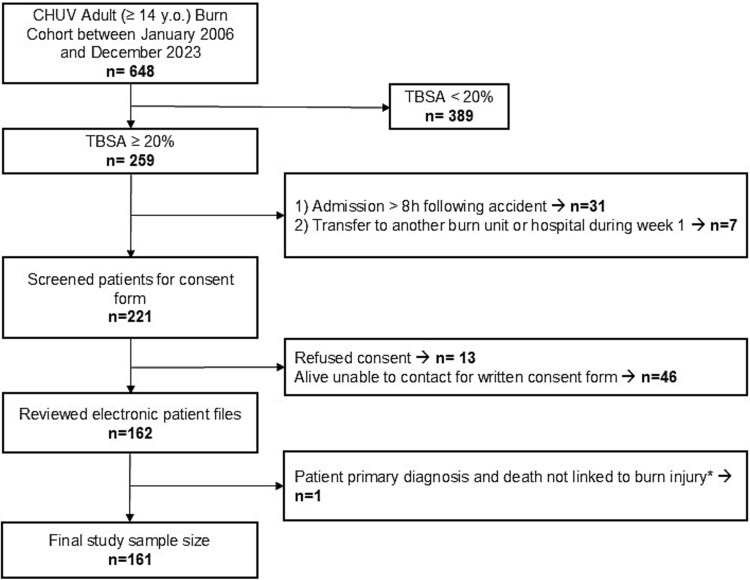
Flowchart Showing the Screening Process of 648 Admitted Patients, Yielding a Final Sample of 161 After Exclusions

A total of 648 patients were screened, of whom 161 were included (Figure 1—flow chart). Patient characteristics are summarized in Table 1. The median TBSA was 38% (IQR 25-65), full-thickness burns 30% (15-55), ABSI score 9 (7-12), and SAPSII score 40 (31-52). The 28-day mortality was 33% (53/161).

**Table 1:** Patients Characteristics in Relation to 28-Day Mortality

Values in median (IQR) or *n* (%)	Total patients (*n* = 161)	Alive (*n* = 108)	Dead (*n* = 53)	*P*-value
Age	48, 31-67	39.5, 25.5-56	68, 48-79	<.001
Male *n*, (%)	115 (71.43%)	83 (76.85%)	32 (60.38%)	.03
TBSA (%)	38, 25-65	30, 22.5-50	67, 35-85,	<.001
Full-thickness burns (%)	30, 15-55	20, 12-40.5	55, 30-80	<.001
Inhalation injury, *n* (%)	101 (62.73%)	62 (57.41%)	39 (73.58%)	.046
SAPS II	40, 31-52	35.5, 29-44	56, 49-68	<.001
ABSI	9, 7-11.5	8, 7-10	12, 10-14	<.001
Total fluid volume administered at 24 h (mL/kg/%TBSA)	3.95, 3.10-5.01	4.13, 3.27-5.20	3.56, 2.76-4.78	.023
Total fluid volume administered at 24 h (mL/kg)	157.03, 92.96-219.09	152, 91.28-205.89	163.77, 98.93-248.94,	.270
Total fluid volume administered at 48 h (mL/kg/%TBSA)	5.84, 4.32-7.19	5.89, 4.98-7.54	4.44, 3.17-6.42	.002
Total fluid volume administered at 48 h (mL/kg)	216.13, 145.69-307.06	218.73, 143.23-307.06,	191.78, 152.93-305.84	.782
Total administered noradrenaline at 24 h (mg)	3.35, 0.1-13.3	1.45, 0-9.65	12.22, 4.59-30.5	<.001
Total administered noradrenaline at 48 h (mg)	8.5, 0.7-32.67	4.3, 0.17-26.99	24.68, 9.30-50.4	<.001
Albumin administration in first 24 h *n*, (%)	59 (36.65%)	34 (31.48%)	25 (47.17%)	.052
> Time to first dose of albumin (when given) (h)	10, 7-17.5	14, 7-18	8, 6-13	.006
> Total administered albumin at 24 h (when given) (g)	40, 20-60	40, 20-60	40, 20-60	.966
> Total administered albumin at 48 h (when given) (g)	90.30, 49.29-120	94.35, 71.3-140	82.44, 35.71-109.91	.159
Minimal serum albumin value in the first 24 h (g/L)	22, 15-28	23, 17-30	19.5, 13-23	<.001
Average UOP in the first 24 h (mL/kg/h)	0.41, 0.24-0.70	0.53, 0.32-0.84	0.16, 0.02-0.27	<.001
RRT within the first 7 days, *n* (%)	20 (13.51%)	11 (10.19%)	9 (22.50%)	.052
AKI within the first 7 days, *n* %	80 (54.05%)	46 (42.59%)	34 (85%)	<.001
Mechanical ventilation duration (day)	6, 1-17.5	11.01, 3-23	1.33, 1-3.4	<.001
ICU length of stay (day)	17, 3-33	25, 15-54	1, 1-4	<.001

Abbreviations: ABSI, Abbreviated Burn Severity Index; AKI, acute kidney injury; IQR, interquartile range; RRT, renal replacement therapy; SAPS II, simplified acute physiology score II; TBSA, total body surface area burned; UOP, urinary output.

Demographic and clinical differences between survivors and non-survivors are reported in Table 1. Non-survivors were significantly older, had larger TBSA burns with a higher proportion of full-thickness burns, more frequently sustained inhalation injuries, and consequently exhibited higher SAPS II and ABSI scores—all of these differences were significant.

The total fluid volume administered was higher in survivors than in non-survivors at 24 h (4.1 vs 3.5 mL/kg/%TBSA; *P* = .023) and 48 h (5.9 vs 4.5 mL/kg/%TBSA; *P* = .002). Non-survivors required higher cumulative doses of noradrenaline at 24 h (12.2 vs 1.45 mg; *P* < .001) and 48 h (24.7 vs 4.3 mg; *P* = .001). Urine output during the first 24 h was lower in non-survivors (0.2 vs 0.5 mL/kg/h; *P* < .001). Acute kidney injury was more common in non-survivors (85% vs 43%; *P* < .001). The use of RRT was higher (22.5% vs 10.2%), although this difference did not reach statistical significance (*P* = .052).

On day 1, minimal serum albumin levels were lower in non-survivors than survivors (19.5 vs 23 g/L; *P* = .004). When administered, albumin administration within the first 24 h was more frequent among non-survivors (47.2% vs 31.5%; *P* = .052) and initiated earlier (median 8 vs 14 h postadmission; *P* = .006). The amount of albumin administered in the first 24 h did not differ significantly between groups (40 vs 40 g; *P* = .966). In univariate analysis, lower albumin levels were associated with higher mortality (OR 0.91; 95% CI, 0.86-0.96; *P* = .001). However, after adjusting for ABSI score, this association was no longer significant (OR 0.99; 95% CI, 0.93-1.06; *P* = .870). Repeating the multivariable analysis after excluding patients who died within the first 24 h, the association remained nonsignificant (OR 0.98; 95% CI 0.91-1.07; *P* = .756).

**Figure 2:**
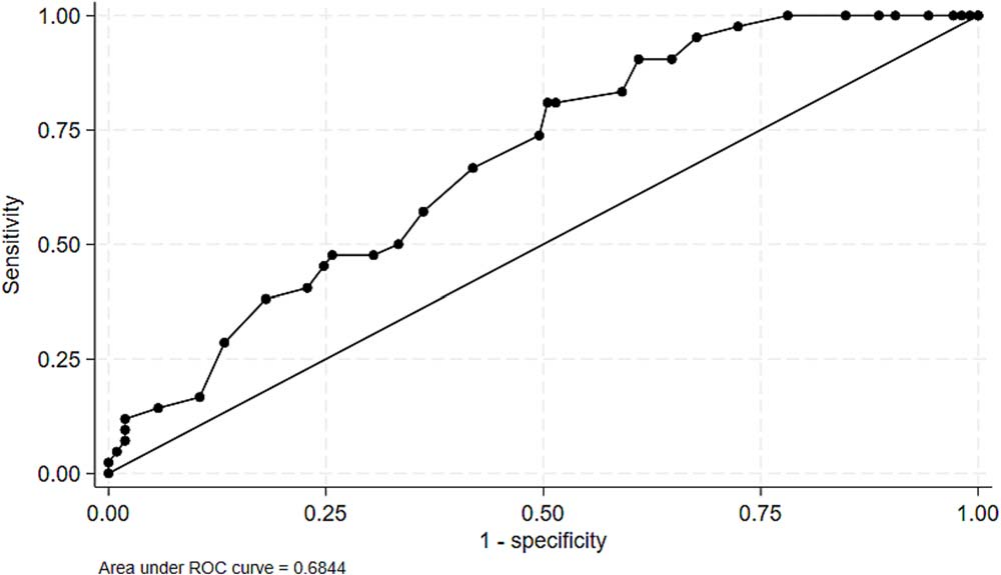
Receiver‑Operating Characteristic (ROC) Curve for Day‑1 Minimal Serum Albumin Level Predicting 28‑day Mortality With an Area Under the Curve (AUC) of 0.684

**Figure 3:**
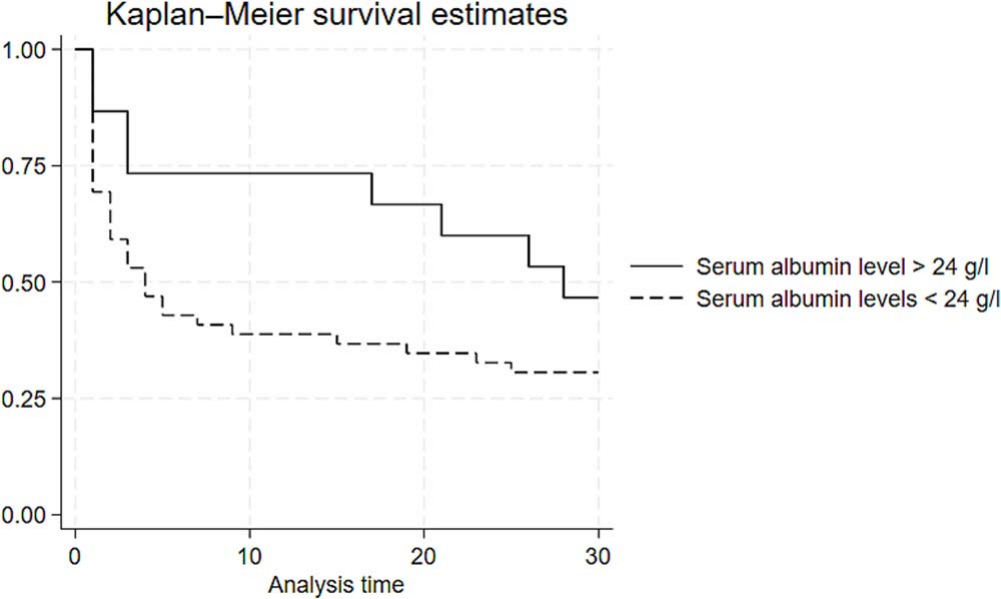
Kaplan–Meier Estimate of 28 Days Survival for Patients With Day‑1 Serum Albumin ≥24 g/L Compared With < 24 g/L

The predictive ability of the minimum serum albumin level for 28-day mortality was poor, with an AUC of 0.684 (95% CI, 0.59-0.77; Figure 2). The optimal cut-off value was 24 g/L, yielding a sensitivity of 81.0% and a specificity of 49.5%. Kaplan–Meier survival analysis (Figure 3) suggested higher mortality in patients with albumin levels below 24 g/L, but this difference was not statistically significant in Cox regression (HR 1.72; 95% CI, 0.79-3.73; *P* = .169).

**Figure 4:**
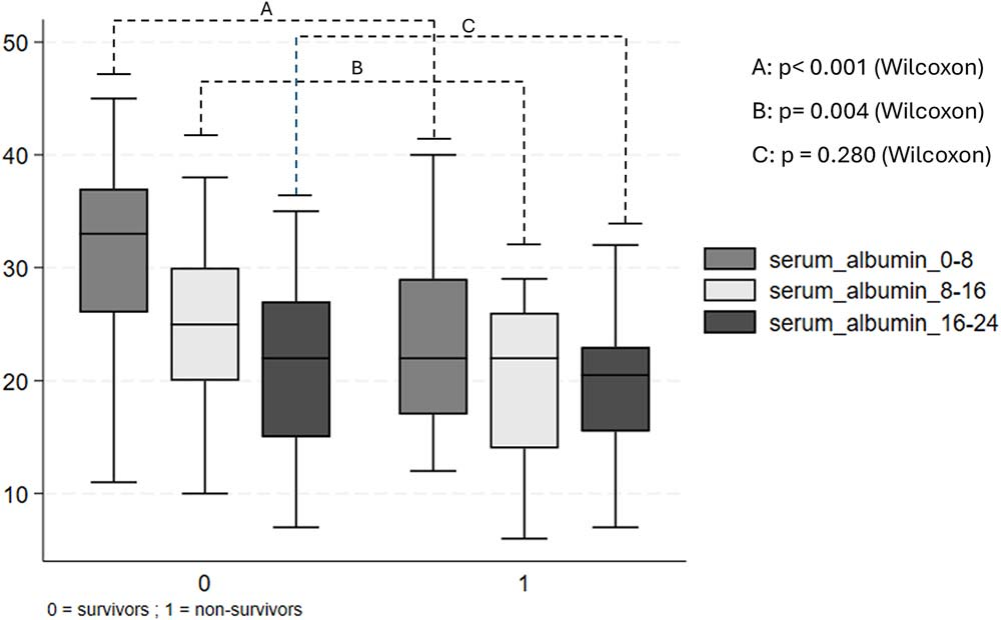
Box‑And‑Whisker Plots Comparing Serum Albumin Levels at 0–8, 8–16, and 16–24 Hours Between Survivors and Non‑Survivors

Figure 4 shows albuminemia values at different time intervals (0-8 h, 8-16 h, and 16-24 h) for survivors and non-survivors. The biggest differences between survivors and non-survivors were observed within the first 8 hours. When analyzing only albumin values from the first 8 h and adjusting for ABSI score, the association with mortality remained nonsignificant (OR 0.94; 95% CI, 0.89-1.00; *P* = .07).

## DISCUSSION

In this cohort of patients with severe burn injuries, hypoalbuminemia within the first 24 h was associated with increased mortality in univariate analysis but was not an independent predictor after adjustment for burn severity.

The results align with those of Eljaiek et al.,^6^ who observed in a smaller cohort (*n* = 56), that hypoalbuminemia was an independent risk factor for organ dysfunction but not for mortality. De Tymowski et al.^9^ examined 73 patients with severe burn injuries having similar TBSA but less extensive full-thickness burns, as well as lower ABSI scores and mortality rate; they reported that only albuminemia measured between admission and the fourth hour was independently associated with 28-day mortality. We were unable to verify these findings due to the absence of albumin measurements within the first 4 h in our cohort. Nevertheless, a multivariate analysis, including only serum albumin values from the first 8 h, also failed to show an association with increased mortality. Aguayo-Becerra et al. studied a larger cohort (*n* = 486), but their patients had considerably less severe burns, with 83.1% sustaining ≤ 30% TBSA and an overall low mortality rate of 7.2%.^5^ Their univariate analysis suggested an association with mortality, but no multivariate analysis was performed. Finally, our results contrast with those of Rafiezadeh et al.^8^ who examined 105 patients with severe burn injuries (TBSA 40%; ABSI score 8.4; mortality 23.8%). Their multivariate analysis identified both the ABSI score and albumin measured 24 h as independent risk factors for mortality. In that study, 19 patients were excluded due to severe comorbidities and albumin administration was not reported.

Some studies used arbitrary cut-off values of 30 g/L^6^ or 20 g/L^5^ for analytical purposes. For studies that determined threshold values, the results are generally consistent with our findings. De Tymowski et al.^9^ identified albumin levels below 23 g/L at 4 h as predictive, while Aguayo-Becerra et al.^5^ reported a mortality risk > 80% for levels below 20 g/L. Shahi et al.^7^ reported thresholds of 35 g/L on admission and 24 g/L at 24 h. The sensitivity of our threshold aligns with these studies, although our specificity was lower (49.52% vs 70%-85%).

To date, this study is among the largest to address this question with 161 patients with TBSA > 20%. Only Aguayo-Becerra et al.^5^ included more patients (*n* = 486), but their cohort was heterogenous and less severely burned (mean TBSA 17.2%) with only 30% of patients sustaining burns > 30%TBSA. Another strength of our study is the severity of burn injuries, reflected by a median TBSA of 38%. This is attributable to the ethics committee’s inclusion criteria, limiting exclusion to individuals who actively refused or could not be contacted, resulting in an overrepresentation of deceased patients. Consequently, this cohort is particularly relevant for assessing mortality prediction. The 28-day mortality rate of 33% is higher than the 10% reported in the ABRUPT study cohort,^19^ which can be partly explained by our inclusion criteria and the greater burn severity in our patients (median TBSA 38% vs 31.6%; full-thickness burns 30% vs 8%). The ABRUPT cohort excluded patients who underwent burn excision within 48 h, died or transitioned to comfort care within 48 h, or had severe comorbidities. As policies on therapeutic withdrawal vary from one institution or country to another, and comorbidities are not always known at admission, we chose a pragmatic approach by including all eligible patients to minimize subjectivity.

Of the 53 non-survivors, 27 died within the first 24 h, and 39 within the first 72 h after admission. Since capillary leakage—and consequently extravascular albumin loss—peaks within 12-24 h after burn injury,^20^^–^^22^ it could be argued that albumin levels in these early deaths may not have had sufficient time to decline fully. However, repeating the analysis after excluding patients who died within the first 24 h did not render the results of the multivariate analysis significant. Moreover, a substantial proportion of patients (36.7% of the cohort and 47.2% of those who died) received albumin within the first 24 h, which may have influenced serum albumin levels. However, this reflects routine practice in most burn centers, where albumin administration is recommended to reduce resuscitation volumes and improve urine output.^13^ Assessing the prognostic value of serum albumin levels in the context of albumin use, which has become standard of care, therefore appears particularly relevant. Finally, it should be noted as a limitation that 27 patients died within the first 24 h, which explains the lower fluid intake observed in the non-survivor group. However, our resuscitation policy follows a maximalist approach during the first 48-72 h. Patients who died prematurely within the first 24 h are therefore considered “non-responders,” rather than individuals for whom care was withdrawn. Accordingly, we believe it is appropriate to include the serum albumin levels of these patients in our analysis. Overall, albumin was not an independent risk factor for mortality but a surrogate marker of severity of illness in this cohort. Not only does this study question the value of albumin levels in predicting prognosis but also raises the question of the relevance of replacing or correcting low serum albumin levels. Ongoing randomized controlled trial and observational study (respectively, NCT04356859 and NCT04264065) may help clarify these questions.

## CONCLUSION

In this large cohort of patients with severe burn injuries, hypoalbuminemia during the first 24 h was not independently associated with an increased 28-day mortality rate. It appears to be only a surrogate marker of burn severity. The predictive value of albumin levels during the first 24 h remains limited, particularly in centers where albumin is administered liberally.

## Notes

Author contributions: Christoph Ils (Conceptualization [equal], Data curation, Formal analysis [lead], Investigation, Methodology, Validation [equal], Writing—original draft [lead], Writing—review & editing [equal]), Tobias Zingg (Conceptualization [supporting], Methodology, Validation, Writing—review & editing [equal]), Mette Berger (Conceptualization, Methodology [supporting], Validation [equal], Writing—original draft [supporting], Writing—review & editing [equal]), Jean-Daniel Rouvé (Conceptualization, Methodology [supporting], Validation, Writing—review & editing [equal]), Lara Montoro (Conceptualization, Data curation, Methodology [supporting], Validation, Writing—review & editing [equal]), Anthony de Buys Roessingh (Conceptualization, Methodology [supporting], Validation, Writing—review & editing [equal]), Karim Al-Dourobi (Validation, Writing—review & editing [equal]), Yves Harder (Validation, Writing—review & editing [equal]), and Olivier Pantet (Conceptualization [equal], Data curation, Formal analysis [supporting], Investigation, Methodology [equal], Project administration, Supervision [lead], Validation [equal], Writing—original draft [supporting], Writing—review & editing [equal])

Funding: This research did not receive any specific grant from funding agencies in the public, commercial, or non-profit sectors.
